# Citizen Science Tick Observations Serve as an Early Warning System for Tick‐Borne Diseases

**DOI:** 10.1111/zph.70045

**Published:** 2026-02-16

**Authors:** Jani Jukka Sormunen

**Affiliations:** ^1^ Department of Biology University of Turku Turku Finland; ^2^ School of Medical, Molecular, and Forensic Sciences Murdoch University Murdoch Western Australia Australia

**Keywords:** borreliosis, crowdsourcing, early warning, Lyme disease, tick‐borne diseases, ticks

## Abstract

**Introduction:**

Tick observation data collected through citizen science is increasingly utilised to map tick‐borne infection risk areas indirectly, that is, based on the rate of tick encounters or occurrence of ticks. However, direct associations between tick observations and Lyme borreliosis (LB) cases have received little attention. In the current study, associations between weekly tick observations and LB cases were studied on a nationwide scale in Finland, in order to determine if tick observations precede cases in a predictable manner, and whether tick observations could be used to predict peaks in cases.

**Methods and Results:**

Nationwide weekly electronic citizen science tick observation data from a tick surveillance website (www.punkkilive.fi/en) and Lyme borreliosis data from the Finnish Institute for Health and Welfare from 2021 to 2023 were utilised in the current study. Negative binomial models were fitted to assess whether tick observations explain variation in LB cases beyond simple seasonality, and to determine if weekly disease cases can be predicted based on tick observation data originating from either humans, pets (dogs & cats) or all sources. Disease cases followed observations with a three to four week lag. Tick observation data were observed to explain variation in LB cases beyond simple seasonality. Models only utilising observations from humans to predict disease cases had the best performance. Finally, differences in the phenology of the two human‐biting tick species present in Finland were observed to influence temporal patterns of observations and LB cases on smaller spatial scales.

**Conclusions:**

This study revealed that LB cases can be predicted utilising citizen science tick observation data. Consequently, crowdsourced tick observation data can be used to predict when peaks in disease cases are to be expected, allowing for specifically targeted awareness campaigns. This, in turn, may lead to symptoms being detected and recognised earlier, allowing for more rapid treatment and fewer sequelae. Guidance on setting up similar models is provided. Actors with access to such data are encouraged to set up similar early warning systems. This increased utility of the data can be leveraged to justify setting up tick observation services, as well as to motivate citizens to participate.

## Impacts

- Crowdsourced tick observation data was associated with diagnosed Lyme borreliosis cases with a lag of 3–4 weeks and explained variation in case numbers beyond simple temperature‐driven seasonality.
- Models combining tick observation data and secondary variables controlling seasonality and spatial variation were able to predict weekly Lyme borreliosis cases on a nationwide scale regardless of observation source (humans, pets or all sources).
- Models on smaller spatial scales functioned with only tick observation and seasonality data and revealed varying temporal patterns driven by different phenologies of local tick species.

## 1 Introduction

Climate change is increasing the worldwide burden of vector‐borne diseases (Watts et al. 2018). In the temperate regions of the Northern Hemisphere, Lyme borreliosis (LB), caused by *Borrelia burgdorferi* sensu lato spirochetes vectored by ticks (Acari: Ixodidae), is the most common vector‐borne disease (Marques et al. 2021; Teh et al. 2023; Wu et al. 2013; Goren et al. 2023). Substantial increases in the disease burden of LB have been observed during the past few decades (Stark et al. 2023). The most common manifestation of LB is the wandering rash, *erythema migrans* (EM). In Finland, LB is most commonly diagnosed based on EM by general practitioners in primary health care. Such diagnoses do not require additional laboratory tests, reducing the time from the detection of symptoms to treatment. An association between delayed treatment of LB (ranging from > 30 days to > 6 weeks) and post‐treatment symptoms has been reported, highlighting the clinical relevance of minimising diagnostic delay (Hirsch et al. 2020; Ljøstad and Mygland 2010). The time delay from the bite of a tick infected with *Borrelia burgdorferi* s.l. to the appearance of EM has commonly been reported as 5–30 days (Trevisan et al. 2020). As such, diagnoses based on EM may be made relatively soon after tick bite. However, knowing to look for symptoms requires knowledge of ticks and symptoms of LB, the level of which is highly varying (Estrada‐Pena et al. 2025). The ability to provide timely bulletins regarding ticks and symptoms of LB during times of high risk could enable earlier detection and treatment, preventing long‐lasting sequelae.

Human LB cases result from the bites of infected ticks. Ticks of the *Ixodes ricinus* species complex, the main transmitters of Lyme borreliosis spirochetes (Gray et al. 2016), reportedly require minimum daily mean temperatures between 4°C and 10°C to be active (Duffy and Campbell 1994; Perret et al. 2000). In climates exhibiting clear seasonality and cold winters, such as Northern Europe, temperatures within a year form a down‐facing parabola, setting a clear limit for tick activity periods with mean temperatures below 4°C roughly from October to early April (Qviller et al. 2014). Consequently, this temperature‐driven seasonality itself explains temporal variation in disease cases to a degree. However, also tick awareness and various factors affecting micro‐scale tick and human activity and movement patterns influence the acquisition of infections. Consequently, data incorporating all these factors, that is, recording specifically when and where humans are contacting ticks, could be more precise for predicting disease cases.

Citizen science has arisen as a useful tool for tick and tick‐borne pathogen research during the past decade (Lernout et al. 2019; Eisen and Eisen 2021; Nieto et al. 2018; Laaksonen et al. 2017; Sormunen et al. 2023; Omazic et al. 2025). It allows for the collection of data sets on ticks and associated pathogens that would be beyond the scope of field surveys in terms of spatial and temporal extent (Laaksonen et al. 2017; Lewis et al. 2018; Porter et al. 2021). Furthermore, citizen science data depicts specifically when and where humans are contacting ticks (Eisen and Eisen 2021). Crowdsourced data has been utilised to project risk areas based on the numbers of reported human‐tick contacts or tick and tick‐borne pathogen occurrence (Nieto et al. 2018; Porter et al. 2021; Bald et al. 2025). However, whether high numbers of tick observations indicate high LB risk depends also on their direct associations with diagnosed LB cases, which have received little attention thus far (Porter et al. 2019). A study conducted in the Northeastern United States found that annual LB numbers could be predicted based on citizen science submissions (Porter et al. 2019). However, there are high correlations between the number of inhabitants, citizen science observations and LB cases (Sormunen et al. 2023; Sun et al. 2025). Associations between tick observations and LB cases on an annual level may mainly describe these relationships. Temporally more precise data are required for assessments of direct associations between observations and LB cases, and for determining whether tick observations precede disease cases in a predictable manner.

In the current study, associations between weekly, electronic citizen science tick observations and LB cases are studied on a nationwide scale in Finland. First, tick observation data is compared to purely seasonal data in order to determine whether observations explain variation—and provide additional value for predictions—beyond seasonality. Second, negative binomial models are fitted to determine how well weekly disease cases can be predicted based on tick observation data originating from either humans, pets (dogs and cats) or all sources. Finally, negative binomial models are fitted on the level of specific hospital districts to observe whether districts with different compositions of human‐biting tick species with different phenologies exhibit different seasonal patterns in tick observations and disease cases.

## 2 Materials and Methods

### 2.1 Lyme Borreliosis and Tick Observation Data

Lyme borreliosis data for 2021–2023 was obtained from the Register for Primary Health Care Visits (Avohilmo), maintained by the Finnish Institute for Health and Welfare (FIHW). Data stored in Avohilmo consists of LB cases diagnosed in municipal health centres by general practitioners, which are mandatory to report to the register. Diagnosis is almost exclusively based on EM infections (Feuth et al. 2020). The LB data was divided by week and hospital district (*n* = 21; Figure S1). For 2021, only data from week 15 onwards was included, since tick observation data was only available after this time.

Tick observation data for 2021–2023 was obtained from a website for reporting tick observations in Finland, Punkkilive (www.punkkilive.fi/en) (Sormunen et al. 2023). The data used in the current analyses (*n* = 298,535) contained the coordinates of tick observations, the date of the observation and from which host (human, pet or nature) the tick was detected. These data were mapped to hospital districts utilising QGIS software (version 3.28 ‘Firenze’) and divided by week, to match LB data.

### 2.2 Statistical Analyses

#### 2.2.1 Variable Selection

**FIGURE 1:**
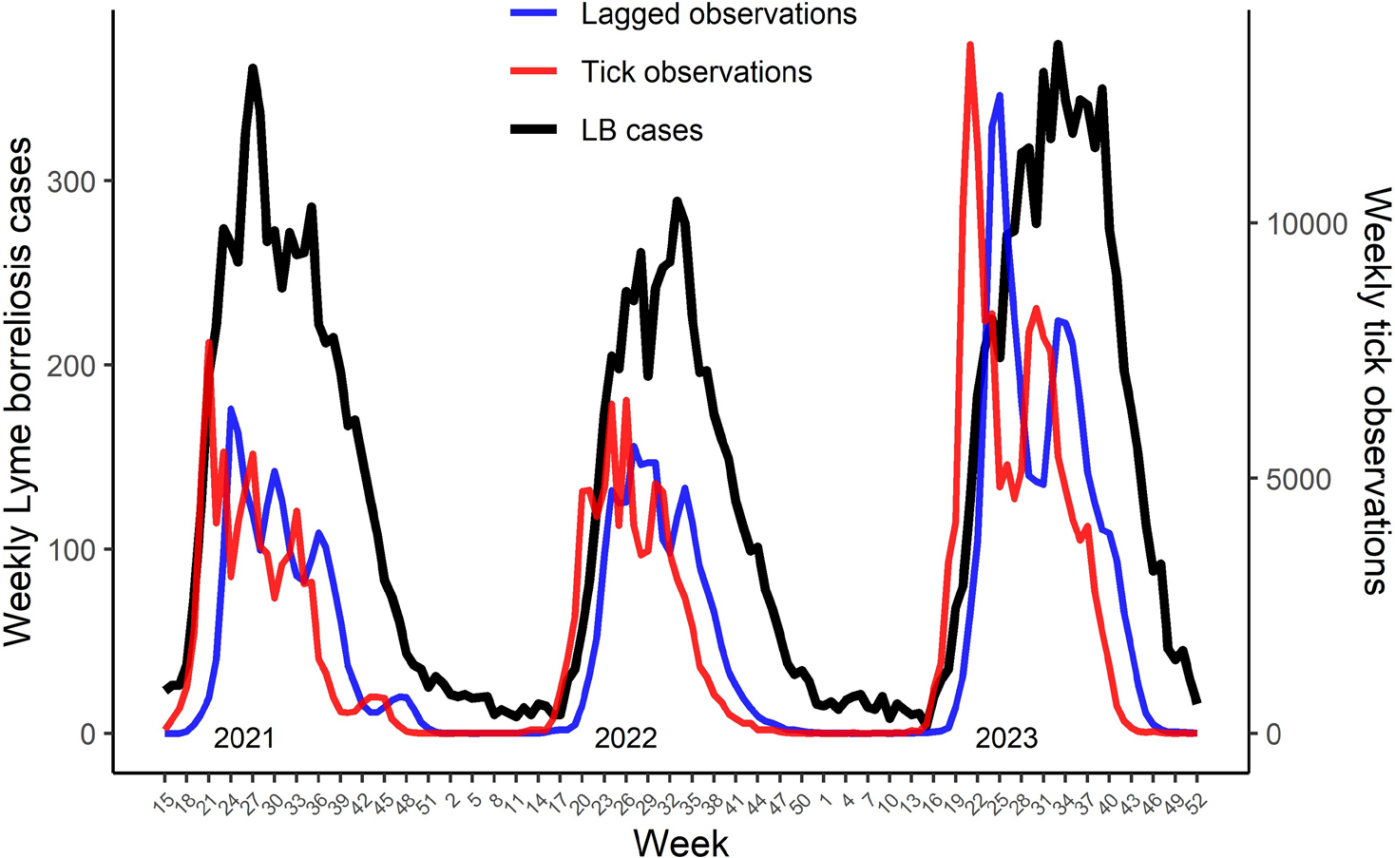
Weekly diagnosed Lyme borreliosis cases and tick observations from 2021 to 2023. The lagged observations used in analyses are also shown (average between 3‐ and 4‐week lags). Please note the different scales of y‐axes.

To determine the most suitable time lag for tick observation data to utilise in analyses (Figure 1), Pearson correlations and AIC, *R* ^2^ values and RMSE from models where disease cases were explained by observation counts with varying lags were calculated (up to 10 weeks) (Table S1).

The population of each hospital district was included as a predictor in models (2021 population census data from Statistics Finland), since the numbers of disease cases and tick observations are strictly linked to the number of inhabitants in a given area [correlations: population vs. LB cases: *r* (21) = 0.94, *p* < 0.0001; population vs. observations: *r* (21) = 0.96, *p* < 0.0001]. However, the districts also have other major differences, including their different geographical locations, which influence, for example, the onset and end of tick activity periods. As such, hospital district was additionally included as a random effect in the models, to account for spatial variation.

In order to account for seasonality, harmonic terms representing baseline annual seasonal variation were calculated and included as predictors in models. For each week of the year (week 1–52), sine and cosine terms were calculated:

$$\sin\;\text{term}=\sin\left(2\pi \frac{\text{week}}{52}\right),\;\cos\;\text{term}=\cos\left(2\pi \frac{\text{week}}{52}\right)$$

These two terms (henceforth ‘seasonal terms’) together represent a full annual cycle and allow the peak of the seasonal curve to occur at any point in the year.

Finally, year (2021–2023) was included as a random effect to account for interannual variation.

#### 2.2.2 Models and Details of Analyses

To determine whether tick observations explain variation in weekly disease case numbers beyond seasonality, two sets of models were fitted: models where tick observations or seasonal terms were the only predictors, and models where all variables apart from tick observations or the seasonal terms were included. A model using residuals of tick observations (after accounting for seasonal terms) together with the seasonal terms was also fitted to test whether tick activity explained unique variation beyond seasonality.

In order to assess how well weekly disease case numbers can be predicted based on tick observation data, models including all the variables were run for the full observation data (*n* = 298,535), as well as subsets where only observations from humans (*n* = 104,965) or pets (*n* = 185,373) were included. For each subset, stepwise models were manually run after the addition of each variable, to observe how they influenced results and fit.

Finally, a selection of hospital districts with different tick species compositions were modelled separately. Hospital districts were classed based on the dominant tick species present, as assessed by crowdsourced data collected in 2015 (Laaksonen et al. 2017). Three districts with only *Ixodes ricinus* present, one district with a highly dominant *I. persulcatus* population (> 90% of samples), and three mixed districts (33%–65% *I. persulcatus* ) were chosen (Table S2). Only one district for *I. persulcatus* was included in analyses since other districts had too low LB case numbers (Table S2). Only tick observations from humans and the seasonal terms were included as predictors in these models.

Since data was divided by week and hospital district, each model utilising all hospital districts had *n* = 2961, whereas hospital district specific models had *n* = 141. LB data as the response variable was utilised as original count data, whereas observation counts and population density were log‐transformed due to high variation in values. Likewise, z‐standardisation was performed on observation counts and population density to aid model convergence. Model fit was assessed based on Akaike Information Criterion (AIC) values, root mean squared error (RMSE; measures the average difference between predicted and observed values) and *R* ^2^ values (proportion of the variation in the response variable explained by the model; marginal *R* ^2^ for fixed effects, conditional R^2^ for fixed + random effects).

All statistical analyses and data manipulation were done using RStudio (build 576). The negative binomial models were fitted using glmer.nb from package *lme4* (Bates et al. 2015). Plots were created using packages *ggplot2* (Wickham 2016) and *patchwork* (Pedersen 2024). Marginal and conditional *R* ^2^ values for models were calculated using package *performance* (Lüdecke et al. 2021).

## 3 Results

Lags of 3 and 4 weeks were observed to be the most associated with disease cases, but they were also nearly identical regarding the measured fit statistics (Figure S2; Table S1). As a compromise between these two lags, the average of their weekly observation numbers was chosen as a predictor for the models. A ratio of roughly one case of LB for every five tick observations from humans was observed (total LB cases: 19078).

**FIGURE 2:**
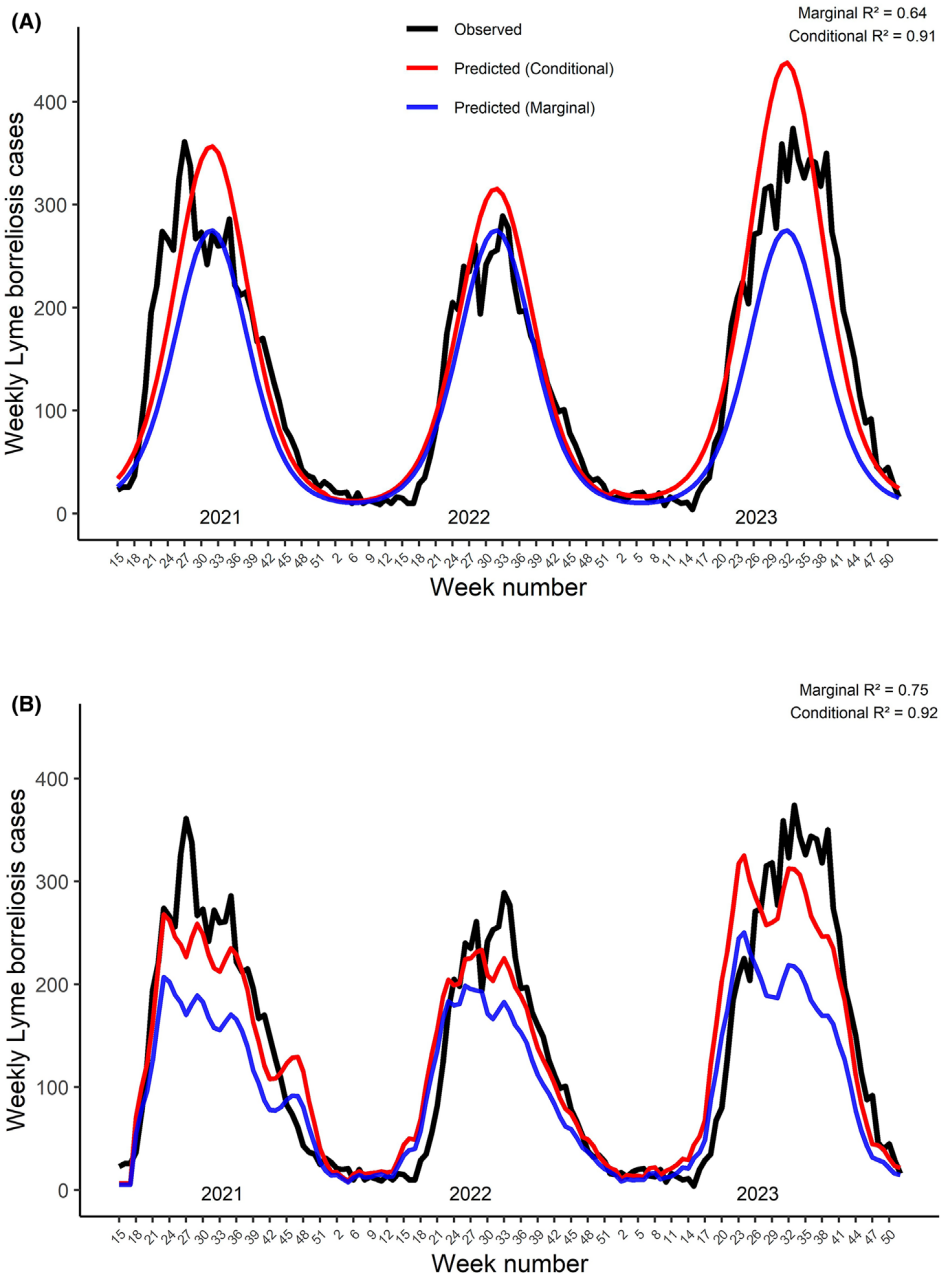
Weekly Lyme borreliosis cases and predictions from the seasonal (A) and tick observation (B) models.

The raw fit of observation counts (AIC = 13,234, RMSE = 13.3, *R* ^2^ = 0.79) surpassed that of the seasonal terms (AIC = 14,424, RMSE = 15.6, *R* ^2^ = 0.43) in models where they were the lone predictors. However, when variables controlling for spatial and temporal variation were added, the variation in disease case numbers explained by the seasonal model increased markedly, as did prediction accuracy (Table 1; Figure 2A). The observation count model achieved somewhat better prediction accuracy, whereas the proportion of variation explained by the fixed terms of the model increased markedly (marginal *R* ^2^ 0.75 vs. 0.64) (Table 1; Figure 2B). Consequently, less variation was explained by random effects in the tick observation model, indicating that tick observation data captures more variation and suffers less from the lack of seasonal data than *vice versa*. Residuals of tick observations were likewise highly significant [estimate = 0.8 ± 0.04 (SE), *z*‐value = 18.7, *p* < 0.0001], further highlighting that tick observations contain unique variation beyond seasonality influencing disease case numbers.

**TABLE 1:** Model fit statistics from negative binomial models utilising crowdsourced tick observations or seasonal data to explain weekly Lyme borreliosis case numbers.

Model	AIC	RMSE	Marginal *R* ^2^	Conditional *R* ^2^
Tick observations only				
Human	13,068	12.8	0.83	
Dog and cat	13,383	13.6	0.75	
All	13,234	13.3	0.79	
Full model				
Human	10,613	4.5	0.74	0.92
Dog and cat	10,794	5.2	0.71	0.92
All	10,754	4.9	0.73	0.93
3‐week lag	10,797	5.0	0.71	0.92
4‐week lag	10,766	5.0	0.71	0.92
Seasonal term vs. tick observations				
Seasonal harmonics	11,092	5.9	0.64	0.91
Tick observations	10,999	5.6	0.75	0.92

**FIGURE 3:**
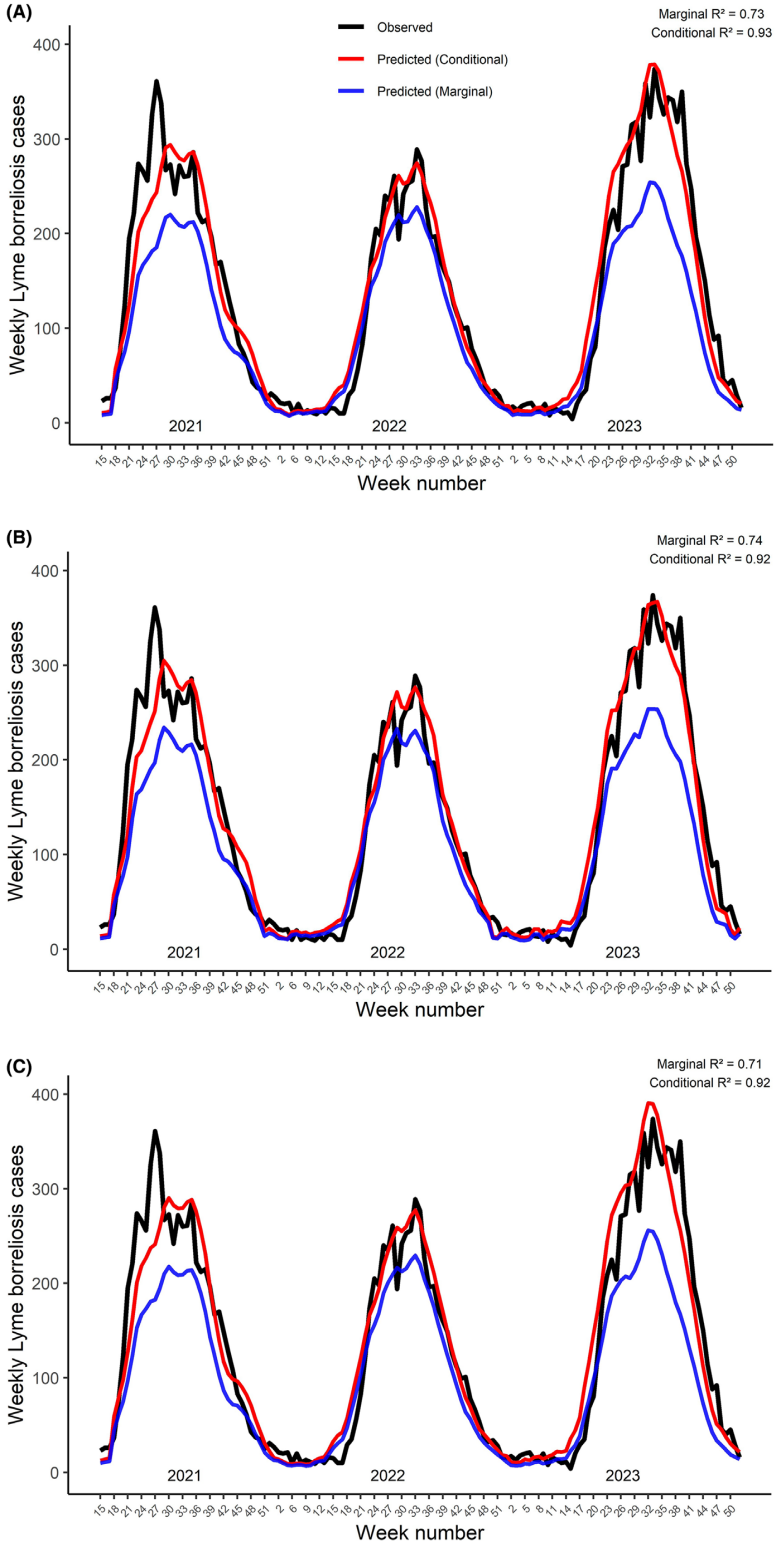
Weekly Lyme borreliosis cases and predictions from full models including all tick observations (A), only observations from humans (B) or only observations from pets (C).

Models based on tick observations from different hosts (humans, pets or all) had roughly similar *R* ^2^ values and fit statistics (Table 1; Figure 3). In general, the models were well able to predict the starts and ends of periods of higher disease case numbers, whereas predictions of individual peaks within the season were less precise. The full model with observations from humans (Figure 3B) had lower RMSE than models with all observations (Figure 3A) or only observations from pets (Figure 3C), indicating the best fit for predicting. Full models with 3‐ and 4‐week lagged count data (using all observations) showed slightly lower *R* ^2^ values and higher RMSE and AIC than the model using the average of the lags, supporting the choice of the average in models (Table 1). Stepwise reports of model and fit statistics for each host group are provided in Table 2 (human data) and Tables S3 and S4 (all observations and pets).

**TABLE 2:** Statistical results from stepwise negative binomial models utilising tick observations from only humans to explain variation in weekly Lyme borreliosis cases.

	Model 1	Model 2	Model 3	Model 4	Full model
	Estimate (SE)	Estimate (SE)	Estimate (SE)	Estimate (SE)	Estimate (SE)
	*z*‐value	*z*‐value	*z*‐value	*z*‐value	*z*‐value
	*p*	*p*	*p*	*p*	*p*
Variables					
Intercept	0.9 (0.03) 33.4 < 0.0001	0.7 (0.02) 29.4 < 0.0001	0.6 (0.02) 28.6 < 0.0001	0.4 (0.2) 2.2 0.03	0.4 (0.2) 2.0 0.05
Lagged observation count	1.4 (0.03) 53.2 < 0.0001	1.3 (0.02) 55.8 < 0.0001	1.4 (0.04) 32.2 < 0.0001	0.9 (0.04) 23.9 < 0.0001	0.9 (0.04) 22.8 < 0.0001
Population density		0.6 (0.02) 32.7 < 0.0001	0.6 (0.02) 27.8 < 0.0001	0.8 (0.2) 4.7 < 0.0001	0.8 (0.2) 4.7 < 0.0001
Sin term			−0.07 (0.04) −1.8 0.07	−0.4 (0.03) −10.8 < 0.0001	−0.4 (0.03) −11.5 < 0.0001
Cos term			0.2 (0.05) 4.4 < 0.0001	−0.3 (0.04) −7.9 < 0.0001	−0.4 (0.04) −8.7 < 0.0001

				Variance	Variance
				Std. Dev.	Std. Dev.
Random effects					
Healthcare district				0.6 0.8	0.6 0.8
Year					0.01 0.1
Model fit statistics					
AIC	13068	12013	11960	10701	10613
RMSE	12.8	7.0	6.8	4.8	4.5
Marginal *R* ^2^	0.83	0.97	0.97	0.74	0.74
Conditional *R* ^2^				0.92	0.92

**FIGURE 4:**
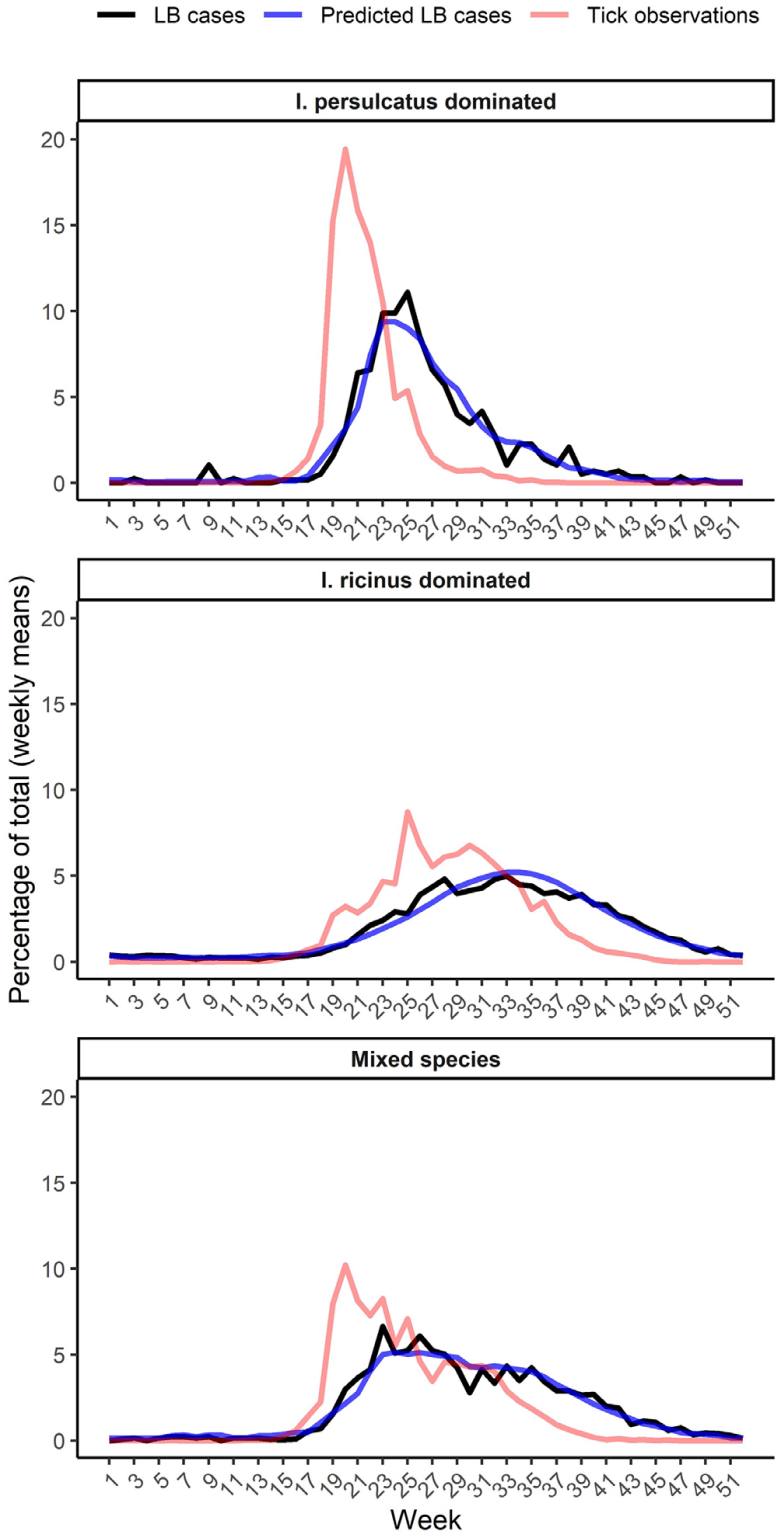
Weekly average percentages of total counts for Lyme borreliosis (‘LB cases’), tick observations (non‐lagged) and predictions from hospital district specific negative binomial models (‘Predicted LB cases’).

The simpler models for specific hospital districts generally obtained high *R* ^2^ values (Table 3). There were distinct trends in seasonality observable across districts dominated by either tick species or having a mix of both species (Figure 4). In the district with a strong *I. persulcatus* population, most of the disease cases were reported between early May and mid‐August (weeks 19–33) and tick observations from late April to late June (weeks 17–25). In *I. ricinus* ‐dominated districts, LB cases were reported more evenly from mid‐May to late November (weeks 20–47) and tick observations from early May to late September (weeks 18–39). Mixed areas formed hybrids between these two, showing increased accumulation of disease cases from early May to mid‐July (weeks 19–29), but continued reporting towards late November. Likewise, tick observations were most numerous from early May to late June (weeks 18–26).

**TABLE 3:** Model fit statistics for hospital district specific negative binomial models utilising tick observations from humans to explain weekly Lyme borreliosis case numbers.

Healthcare district^a^	Tick category	AIC	RMSE	Normalised RMSE^b^	Marginal *R* ^2^
NO	*I. persulcatus* dominated	447	2.2	0.34	0.99
HUS	*I. ricinus* dominated	1110	21.1	0.45	0.99
SF	*I. ricinus* dominated	907	8.4	0.46	0.99
KYM	*I. ricinus* dominated	617	3.0	0.46	0.94
NS	Mixed	610	3.5	0.49	0.98
P	Mixed	586	2.8	0.48	0.99
V	Mixed	424	1.5	0.62	0.86

^a^ Healthcare district abbreviations listed in Figure S1.

^b^ Deviance‐based normalisation.

## 4 Discussion

Tick observations lagged by 3 or 4 weeks were most associated with LB cases, with cases correspondingly following 3–4 weeks after tick observations. This observation is in line with the reported time delay from the bite of an infected tick to the appearance of EM, 5–30 days (Trevisan et al. 2020). This indicates that high numbers of tick observations also signify high risk for LB infections. Consequently, citizen science tick observations may be used to identify the most impactful times for awareness campaigns, as well as to alert healthcare professionals to watch for symptoms.

The progress of the seasons is highly linked to the activity of ticks at northern latitudes (Qviller et al. 2014). Consequently, the model utilising only seasonal terms and controlling for spatial and temporal variation was able to predict disease cases reasonably well. However, tick data was observed to contain information for explaining variation in disease cases including and beyond seasonality. Tick observation data incorporates several different factors into a single value, including not only the progress of the seasons but also micro‐scale differences in human and tick activity. In the end, seasonality mostly depicts when tick activity is to be expected, whereas tick observation data is directly showing when people are contacting ticks within this activity season.

Full models utilising observations from only humans produced the best predictions, highlighting that especially human‐derived tick reports are associated with diagnosed LB cases. Previous clinical studies have estimated that between 1.4% and 5% of tick bites lead to LB (Hofhuis et al. 2017; Wilhelmsson et al. 2016). However, in this study, a much higher ratio of 5:1 was observed between tick observations from humans and diagnosed LB cases. This indicates that roughly one case of LB is reported for every five reported tick contacts. While this data does not show causation like the clinical studies, whether ratios between observations and disease cases are similar in other countries should be assessed. In any case, differences between the full models were relatively small, indicating that most forms of citizen science data can be utilised for predicting human disease cases. Regardless of whether the ticks have been observed from humans or pets, high numbers of observations indicate that many ticks are active in the nature, which in turn translates into increased risk of infection for humans.

Models made on the hospital district level revealed differences in seasonality for both tick observations and disease cases. This was expected, as studies have shown phenological differences between the two human‐biting tick species present in Finland (Laaksonen et al. 2017). *Ixodes persulcatus* are active from snowmelt (March–April) to the end of June, after which the shortening of day length seems to trigger the cessation of their activity (Pakanen et al. 2020; Sormunen et al. 2020, 2025). In contrast, the activity of *I. ricinus* continues to September–October, with peaks in nymph activity commonly recorded in August and September (Pakanen et al. 2020; Sormunen et al. 2020). These differences are not due to weather conditions—for example, September mean temperatures are +10°C in Oulu, a city clearly dominated by *I. persulcatus* and showing very limited tick activity beyond June (Sormunen et al. 2025). These phenological differences are mirrored in the disease cases and tick observations. In areas dominated by *I. persulcatus* , most of the disease cases were reported between early May and mid‐August, corresponding to expected high tick activity from April to the end of June. In areas dominated by *I. ricinus* , disease cases were reported more equally from mid‐May to late November. This corresponds to tick activity from late April to late September, the observed main activity period for *I. ricinus* locally (Sormunen et al. 2025). Finally, districts with both tick species were a mix between dominated areas, with a higher contribution towards total disease cases between May and July, but continued reporting into November. This indicates that during spring/early summer both tick species are active locally, leading to higher disease case numbers, followed by lower numbers when *I. persulcatus* activity decreases after June. Consequently, tick phenology appears to have an observable impact on tick observations and LB cases.

Finally, for making predictions of future disease cases, variation across years cannot naturally be included. However, the inclusion of year seemed to have a minor effect on model performance. As such, tick observation counts together with a seasonal term, population density data and variables accounting for spatial variation can be used to predict disease cases 3–4 weeks in the future. However, it might be beneficial to run the models for smaller areas individually to reduce the impact of spatial variation, in which case only tick observation data and a seasonal term are required. It should be noted that the seasonal terms require prior data to capture the annual trends in LB cases. However, at spatial scales where variation due to geography and climate are smaller, pure tick observation data alone may provide usable predictions. In addition to these, there are also some additional considerations regarding such models:

1. Determination of a locally suitable time lag. Testing of different time lags should be done for different data sets, as there may be differences across nations in awareness and typical time lags in diagnosis. For example, participants from Finland were recently observed to have high awareness regarding ticks and tick‐borne pathogens in a Europe‐wide study, which may contribute towards earlier diagnoses of LB (Estrada‐Pena et al. 2025). Furthermore, different diseases may have different time lags from tick bite to manifestation and treatment.
2. Determination of a locally suitable seasonal term. At northern latitudes, activity of human‐biting tick species is limited to roughly between April and October. As such, a seasonal term with a single peak is suitable for capturing the general annual trend, showing increasing tick activity with increasing temperatures until roughly July, then a decreasing trend towards the end of the year. For other climates, this may need to be adjusted by adding more seasonal terms.
3. Addition of tick species specific data. Tick species have different phenology and biology, which influence when humans come into contact with them. For example, certain species have endophilic and exophilic life stages, in which case only the activity of the exophilic life stages influences risk (Földvári et al. 2016). Likewise, for other species, the occurrence of different life stages may be sequential (Waudby and Petit 2007) (rather than mostly concurrent, as for *I. ricinus* and *I. persulcatus* ). In these cases, different seasonal terms may be needed. Tick experts should be consulted to assess relevant variables when creating such models.

In conclusion, the present study highlights that crowdsourced tick observation data are associated with diagnosed LB cases with a lag of 3–4 weeks. Weekly borreliosis cases can be predicted on a nationwide scale utilising sufficiently lagged observation data and secondary variables accounting for local human population density and seasonality, as well as controlling for spatial variation. However, more locally, even a model combining just tick observations and a seasonal variable provided utilisable predictions of disease cases. Early detection and treatment of LB is of paramount importance to prevent long‐lasting sequelae (Hirsch et al. 2020). Citizen science tick observation data can be used to predict when peaks in disease cases are to be expected, allowing for specifically targeted tick and tick‐borne disease awareness campaigns. This, in turn, may lead to symptoms being detected and recognised earlier, allowing for more rapid treatment and fewer sequelae. Actors with access to tick citizen science data are encouraged to set up such early warning systems against LB and potentially other tick‐borne diseases. Likewise, this increased utility of the data can be leveraged to justify setting up tick observation services, as well as to motivate citizens to participate.

## Funding

This work was supported by the Research Council of Finland (grant number 360177). The Punkkilive website is maintained by Pfizer Oy Finland.

## Ethics Statement

The author has nothing to report.

## Conflicts of Interest

The author declares no conflicts of interest.

## Supporting information


**Figure S1:** Map of healthcare districts.
**Figure S2:** Cross‐correlation analysis for Lyme borreliosis cases and tick observations with different time lags (in weeks).
**Table S1:** Fit statistics for negative binomial models predicting Lyme borreliosis cases based on citizen science tick observations with different lags (weeks).
**Table S2:** Proportion of *Ixodes persulcatus* in tick collection samples, crowdsourced tick observations and diagnosed Lyme borreliosis cases by healthcare districts.
**Table S3:** Stepwise results of statistical analyses for negative binomial models utilising all available observations.
**Table S4:** Stepwise results of statistical analyses for negative binomial models utilising observations from pets.

## Data Availability

The data that support the findings of this study are available from the corresponding author upon reasonable request.
