# Supplementary material for: Citizen Science Tick Observations Serve as an Early Warning System for Tick‐Borne Diseases
Source: Zoonoses Public Health. 2026 Feb 16;73(3):234–43. doi: 10.1111/zph.70045 (PMC13053620; doi:10.1111/zph.70045)
Supplement: Supplementary file 1 — Figure S1: Map of healthcare districts. Figure S2: Cross‐correlation analysis for Lyme borreliosis cases and tick observations with different time lags (in weeks). Table S1: Fit statistics for negative binomial models predicting Lyme borreliosis cases based on citizen science tick observations with different lags (weeks). Table S2: Proportion of Ixodes persulcatus in tick collection samples, crowdsourced tick observations and diagnosed Lyme borreliosis cases by healthcare districts. Table S3: Stepwise results of statistical analyses for negative binomial models utilising all available observations. Table S4: Stepwise results of statistical analyses for negative binomial models utilising observations from pets. [file ZPH-73-234-s001.docx]

Citizen science tick observations serve as an early warning system for tick-borne diseases

Jani J. Sormunen

**Supporting Information**

Contents

[Figure S1. Map of healthcare districts. 2](#_Toc221157950)

[Figure S2. Cross-correlation analysis for Lyme borreliosis cases and tick observations with different time lags (in weeks). 3](#_Toc221157951)

[Table S1. Fit statistics for negative binomial models predicting Lyme borreliosis cases based on citizen science tick observations with different lags (weeks). 3](#_Toc221157952)

[Table S2. Proportion of *Ixodes persulcatus* in tick collection^a^ samples, crowdsourced tick observations and diagnosed Lyme borreliosis cases by healthcare districts. 4](#_Toc221157953)

[Table S3. Stepwise results of statistical analyses for negative binomial models utilizing all available observations. 5](#_Toc221157954)

[Table S4. Stepwise results of statistical analyses for negative binomial models utilizing observations from pets. 6](#_Toc221157955)

[References 6](#_Toc221157956)

# Figure S1. Map of healthcare districts.


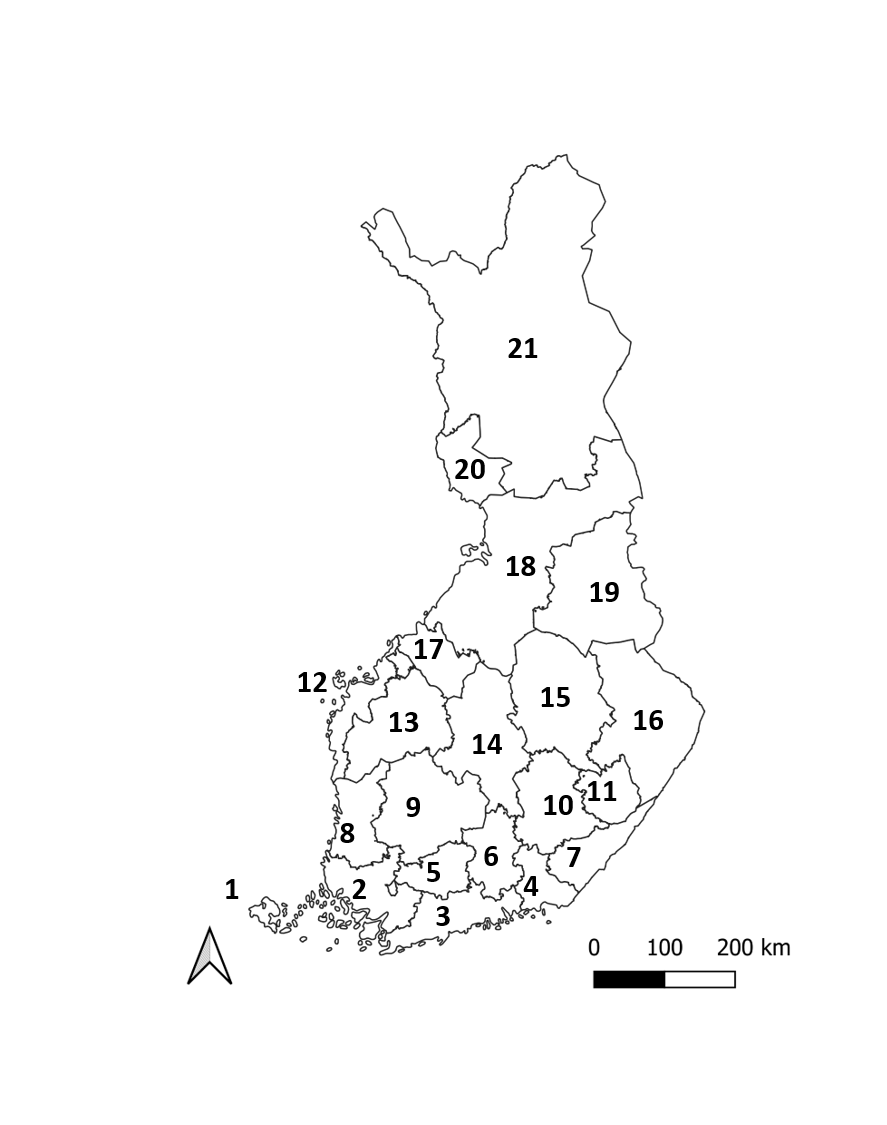


Figure S1. Map of healthcare districts. Districts: 1 = ÅL, Åland; 2 = SF, Southwest Finland hospital district; 3 = HUS, Helsinki and Uusimaa hospital district; 4 = KYM, Kymenlaakso hospital district; 5 = KH, Kanta-Häme hospital district; 6 = PH; Päijät-Häme hospital district; 7 = SK, South Karelia hospital district; 8 = S, Satakunta hospital district; 9 = P, Pirkanmaa hospital district; 10 = SS, South Savo hospital district; 11 = IS, Itä-Savo hospital district; 12 = V, Vaasa hospital district; 13 = SO, South Ostrobothnia hospital district; 14 = CF, Central Finland hospital district, 15 = NS, North Savo hospital district; 16 = NK, North Karelia hospital district; 17 = CO, Central Ostrobothnia hospital district; 18 = NO, North Ostrobothnia hospital district; 19 = KAI, Kainuu hospital district; 20 = LP, Länsi-Pohja hospital district, 21 = L, Lappi hospital district.

# Figure S2. Cross-correlation analysis for Lyme borreliosis cases and tick observations with different time lags (in weeks).


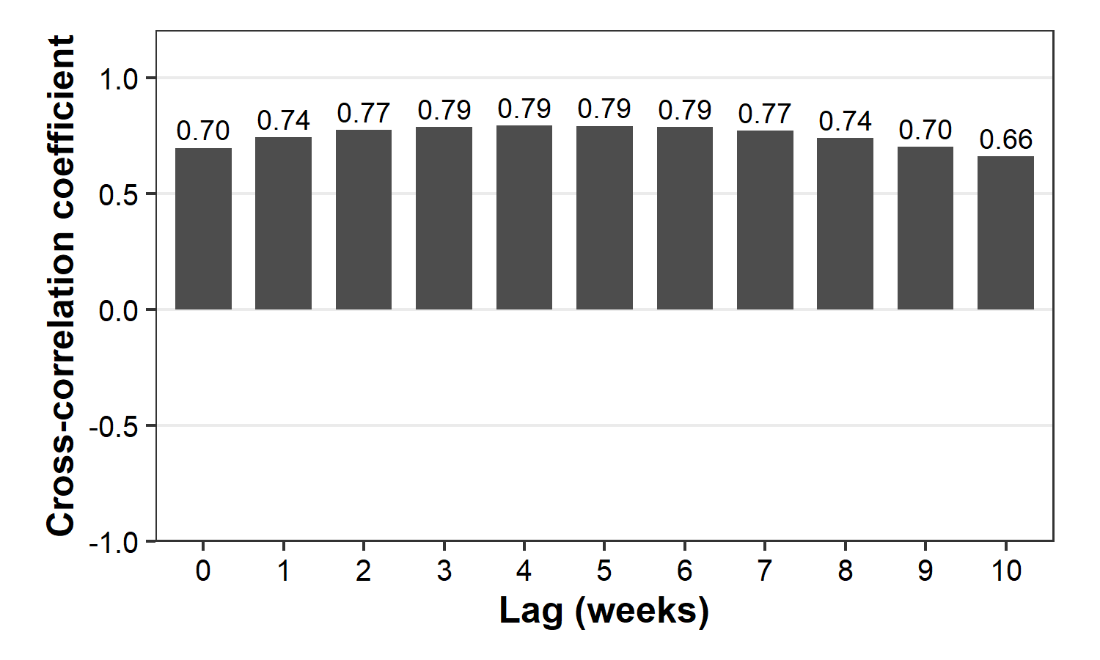


# Table S1. Fit statistics for negative binomial models predicting Lyme borreliosis cases based on citizen science tick observations with different lags (weeks).

| Observation lag | AIC | Marginal R^2^ | RMSE |
| --- | --- | --- | --- |
| 3 | 13300 | 0.77 | 13.6 |
| 4 | 13310 | 0.76 | 13.6 |
| 2 | 13382 | 0.74 | 13.7 |
| 5 | 13511 | 0.71 | 13.8 |
| 1 | 13515 | 0.71 | 13.9 |
| 6 | 13760 | 0.65 | 14.1 |
| 7 | 14104 | 0.54 | 14.4 |
| 8 | 14383 | 0.43 | 14.7 |
| 9 | 14608 | 0.33 | 15.0 |
| 10 | 14773 | 0.27 | 15.3 |

# Table S2. Proportion of *Ixodes persulcatus* in tick collection^a^ samples, crowdsourced tick observations and diagnosed Lyme borreliosis cases by healthcare districts.

| Healthcare district | Proportion of *I. persulcatus*^a^ | Tick observations | Lyme borreliosis cases | Chosen for analysis? |
| --- | --- | --- | --- | --- |
| PH | 0.07 | 10181 | 495 |  |
| NO | 0.92 | 21730 | 574 | *I. persulcatus* |
| SS | 0.08 | 9040 | 329 |  |
| KAI | 0.68 | 3409 | 16 |  |
| SF | 0 | 32709 | 3136 | *I. ricinus* |
| SO | 0.33 | 4265 | 169 |  |
| HUS | 0 | 66684 | 7459 | *I. ricinus* |
| NS | 0.33 | 17925 | 869 | Mixed |
| S | 0 | 9672 | 666 |  |
| CO | 0.94 | 2810 | 76 |  |
| P | 0.60 | 17541 | 826 | Mixed |
| KH | 0.04 | 9251 | 346 |  |
| KYM | 0 | 10634 | 951 | *I. ricinus* |
| ÅL | 0 | 2046 | 635 |  |
| L | 0.44 | 1097 | 43 |  |
| NK | 0.35 | 13959 | 472 |  |
| V | 0.66 | 7369 | 296 | Mixed |
| LP | 0.96 | 3894 | 38 |  |
| CF | 0.02 | 13435 | 896 |  |
| SK | 0.002 | 9745 | 650 |  |
| IS | 0.02 | 3316 | 130 |  |

^a^Data from Laaksonen et al. 2017.

# Table S3. Stepwise results of statistical analyses for negative binomial models utilizing all available observations.

|  | **Model 1** | **Model 2** | **Model 3** | **Model 4** | **Full model** |
| --- | --- | --- | --- | --- | --- |
| **VARIABLES** | Estimate(SE)  z-value  p-value | Estimate(SE)  z-value  p-value | Estimate(SE)  z-value  p-value | Estimate(SE)  z-value  p-value | Estimate(SE)  z-value  p-value |
| Intercept | 0.9 (0.03)  30.9  <0.0001 | 0.7 (0.02)  27.8  <0.0001 | 0.6 (0.02)  25.7  <0.0001 | 0.3 (0.2)  1.6  0.1 | 0.3 (0.2)  1.5  0.13 |
| Lagged observation count | 1.5 (0.03)  47.9  <0.0001 | 1.3 (0.03)  47.6  <0.0001 | 1.2 (0.05)  23.0  <0.0001 | 0.9 (0.04)  19.7  <0.0001 | 0.8 (0.04)  18.7  <0.0001 |
| Population density |  | 0.5 (0.02)  27.6  <0.0001 | 0.6 (0.02)  25.3  <0.0001 | 0.8 (0.2)  4.4  <0.0001 | 0.8 (0.2)  4.4  <0.0001 |
| Sin term |  |  | -0.3 (0.04)  -7.0  <0.0001 | -0.5 (0.03)  -15.4  <0.0001 | -0.5 (0.03)  -16.1  <0.0001 |
| Cos term |  |  | -0.06 (0.05)  -1.2  0.22 | -0.5 (0.04)  -11.4  <0.0001 | -0.5 (0.04)  -12.3  <0.0001 |
| **RANDOM EFFECTS** |  |  |  | Variance  Std. Dev. | Variance  Std. Dev. |
| Healthcare district |  |  |  | 0.7  0.8 | 0.7  0.8 |
| Year |  |  |  |  | 0.01  0.1 |
| **MODEL FIT STATISTICS** |  |  |  |  |  |
| AIC | 13234 | 12412 | 12355 | 10792 | 10754 |
| RMSE | 13.3 | 8.7 | 8.2 | 5.2 | 4.9 |
| Marginal R^2^ | 0.79 | 0.94 | 0.94 | 0.73 | 0.73 |
| Conditional R^2^ |  |  |  | 0.93 | 0.93 |

# Table S4. Stepwise results of statistical analyses for negative binomial models utilizing observations from pets.

|  | **Model 1** | **Model 2** | **Model 3** | **Model 4** | **Full model** |
| --- | --- | --- | --- | --- | --- |
| **VARIABLES** | Estimate(SE)  z-value  p-value | Estimate(SE)  z-value  p-value | Estimate(SE)  z-value  p-value | Estimate(SE)  z-value  p-value | Estimate(SE)  z-value  p-value |
| Intercept | 0.9 (0.03)  33.2  <0.0001 | 0.7 (0.02)  30.6  <0.0001 | 0.7 (0.03)  27.0  <0.0001 | 0.3 (0.2)  1.6  0.11 | 0.3 (0.2)  1.5  0.14 |
| Lagged observation count | 1.5 (0.03)  46.8  <0.0001 | 1.3 (0.03)  45.4  <0.0001 | 1.0 (0.03)  19.0  <0.0001 | 0.7 (0.04)  18.4  <0.0001 | 0.7 (0.04)  17.5  <0.0001 |
| Population density |  | 0.5 (0.02)  26.1  <0.0001 | 0.6 (0.02)  26.2  <0.0001 | 0.8 (0.2)  4.4  <0.0001 | 0.8 (0.2)  4.4  <0.0001 |
| Week (residuals) |  |  | -0.5 (0.04)  -10.7  <0.0001 | -0.6 (0.03)  -17.8  <0.0001 | -0.6 (0.03)  -18.6  <0.0001 |
| Week^2 (residuals) |  |  | -0.3 (0.06)  -4.7  <0.0001 | -0.6 (0.04)  -13.6  <0.0001 | -0.6 (0.04)  -14.5  <0.0001 |
| **RANDOM EFFECTS** |  |  |  | Variance  Std. Dev. | Variance  Std. Dev. |
| Healthcare district |  |  |  | 0.8  0.9 | 0.8  0.9 |
| Year |  |  |  |  | 0.01  0.1 |
| **MODEL FIT STATISTICS** |  |  |  |  |  |
| AIC | 13383 | 12610 | 12513 | 10836 | 10794 |
| RMSE | 13.6 | 9.3 | 8.5 | 5.4 | 5.2 |
| Marginal R^2^ | 0.75 | 0.91 | 0.92 | 0.71 | 0.71 |
| Conditional R^2^ |  |  |  | 0.92 | 0.92 |

# References

1. Laaksonen M, Sajanti E, Sormunen JJ, Penttinen R, Hänninen J, Ruohomäki K, et al., "Crowdsourcing-based nationwide tick collection reveals the distribution of *Ixodes ricinus* and *I. persulcatus* and associated pathogens in Finland," *Emerg Microbes Infec,* vol. 6, p. e31, 2017.
